# Crystal Structure of Ribosome-Inactivating Protein Ricin A Chain in Complex with the C-Terminal Peptide of the Ribosomal Stalk Protein P2

**DOI:** 10.3390/toxins8100296

**Published:** 2016-10-13

**Authors:** Wei-Wei Shi, Yun-Sang Tang, See-Yuen Sze, Zhen-Ning Zhu, Kam-Bo Wong, Pang-Chui Shaw

**Affiliations:** Centre for Protein Science and Crystallography, School of Life Sciences, The Chinese University of Hong Kong, Shatin, N.T., Hong Kong, China; Shiww@cuhk.edu.hk (W.-W.S.); samtys0910@gmail.com (Y.-S.T.); seeyuen123@gmail.com (S.-Y.S.); janet.chuk@gmail.com (Z.-N.Z.); kbwong@cuhk.edu.hk (K.-B.W.)

**Keywords:** Ricin, ribosome-inactivating protein, ribosomal P stalk protein, ribosome

## Abstract

Ricin is a type 2 ribosome-inactivating protein (RIP), containing a catalytic A chain and a lectin-like B chain. It inhibits protein synthesis by depurinating the N-glycosidic bond at α-sarcin/ricin loop (SRL) of the 28S rRNA, which thereby prevents the binding of elongation factors to the GTPase activation center of the ribosome. Here, we present the 1.6 Å crystal structure of Ricin A chain (RTA) complexed to the C-terminal peptide of the ribosomal stalk protein P2, which plays a crucial role in specific recognition of elongation factors and recruitment of eukaryote-specific RIPs to the ribosomes. Our structure reveals that the C-terminal GFGLFD motif of P2 peptide is inserted into a hydrophobic pocket of RTA, while the interaction assays demonstrate the structurally untraced SDDDM motif of P2 peptide contributes to the interaction with RTA. This interaction mode of RTA and P protein is in contrast to that with trichosanthin (TCS), Shiga-toxin (Stx) and the active form of maize RIP (MOD), implying the flexibility of the P2 peptide-RIP interaction, for the latter to gain access to ribosome.

## 1. Introduction

Ricin is a well-known type 2 ribosome-inactivating protein (RIP) discovered in the late 19th century in the seeds of *Ricinus communis*. It agglutinates red blood cells and is one of the most potent and lethal substances known [1]. Ricin hydrolyzes the *N*-glycosidic bond at adenine−4324 in the 28S rRNA of eukaryotic ribosomes [2]. This adenine is located at a GAGA hairpin within the sarcin/ricin loop [3], which is highly conserved in all large ribosomal subunits and is essential for the proper assembly of the functional core of the large subunit [4]. In eukaryotes, removal of the specific adenine hinders the elongation factor 1-dependent binding of aminoacyl-tRNA and GTP-dependent binding of elongation factor (EF) 2 to ribosome. In prokaryotes, damaged ribosomes do not bind EF-Tu or EF-G. As a result, protein synthesis is arrested at the elongation step [5].

Ricin is more active on eukaryotic ribosome compared to naked 28S rRNA. The depurination activity for intact ribosome is over 80,000-fold higher than naked 28S rRNA, suggesting that the presence of ribosomal proteins facilitating the rRNA depurination by ricin [6]. These observations lead to a postulation that the interaction with ribosomal proteins is essential for RIPs to exert the conserved *N*-glycosidase activity. To date, it has been demonstrated that ricin A chain (RTA) interacts with the mammalian L9 and L10e (=P0) ribosomal proteins by crosslinking-trypsin digestion analysis [7] and RTA fails to interact with ribosome mutants lacking P1 or P2 in surface plasmon resonance interaction analysis [8]. RTA-P protein interaction is essential for ribosome inactivating action as yeast mutants with deletion of P1 and/or P2 have less depurination [8]. In eukaryotic ribosome, P proteins are located at the stalk and is composed of a pentameric complex of acidic ribosomal proteins, with one P0 and two P1 and P2, all of which possess a conserved amino acid sequence rich in acidic residues in their C-termini [9].It was also suggested that RTA binds to human ribosomal stalk protein P2 both in vitro and in vivo [10]. The RIP-binding site in P2 proteins has been mapped to eleven highly conserved C-terminal residues SDDDMGFGLFD (C11-P) and peptides corresponding to this sequence are found to be involved in stalk activity [11] and interacts with several RIPs, including trichosanthin (TCS) [12,13], the active form of maize RIP (MOD) [14] and Shiga toxin (Stx) 1 [15,16].

Our group has previously revealed the crystal structure of TCS-C11-P complex at 2.2 Å and identified the three basic residues K173, R174 and K177 in TCS forming charge–charge interactions with the acidic DDD motif while a hydrophobic pocket lined by F166, L188 and L215 accommodates the LF motif [17]. Furthermore, based on our NMR structure of the stalk protein complex of P1/P2 heterodimer and biochemical analyses, we demonstrated that the flexible C-terminal tail of eukaryotic ribosome stalk can form an arm-like structure and extend with a radius up to ~125 Å [18,19,20], hinting that the complex can help recruiting RIPs towards the core of the ribosome upon RIP-P protein interaction. Apart from interacting with RIPs, several studies have reported that the conserved C-terminal motif of ribosomal P proteins is crucial for binding translation factors or interacting with elongation factors in bacterial [21,22], archaeal [11] and eukaryotic ribosomes [23]. All the experimental evidence suggest TCS or other RIPs might hijack the ribosomal stalk proteins by binding to their conserved C-termini to gain access to the SRL of rRNA [24].

RTA and TCS maintain a homologous structure topology and both have been reported to interact with the conserved C-terminus of P proteins. However, they share low similarity in amino acid sequences. Therefore, to decipher the molecular mechanism of RTA-P protein interaction will provide more insights on the interaction mode between RIPs and P proteins. Recently, four positively charged arginines R189, R193, R234 and R235 in RTA were suggested to be important in RIP-stalk binding [25], implying that RTA might adopt same interaction mode to binding to the conserved C-terminal tail of P proteins. In order to clarify the nature of binding between RTA and ribosomal stalk, we crystallized and solved the structure of RTA and the conserved C-terminal nonamer of the P-proteins. Here, we show that RTA adopts a novel binding mode to interact with the P protein via hydrophobic interactions rather than electrostatic interactions. Based on our structural analyses and comparison of the putative P2 binding pocket of TCS, RTA, Stx and MOD, we conclude that P protein may adopt different orientations when interacting with RIPs.

## 2. Results

### 2.1. RTA Interacts with the Conserved C-Terminal Region of Acidic Ribosomal Stalk Protein P

**Figure 1:**
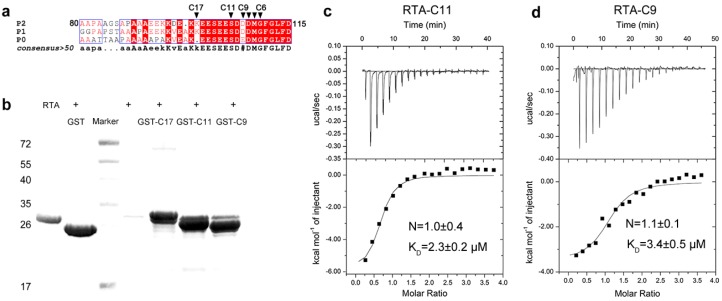
In vitro interaction assays of RTA and P2. (**a**) The C-terminal consensus sequence in the ribosomal P protein family. The sequence alignment was prepared by Multalin online program (http://multalin.toulouse.inra.fr/multalin/). The last residues of the C-terminus sequence of P2 are marked with black triangle; (**b**) GST pull-down assay of C-terminal truncations of P2 and RTA. Lane 1 represents the RTA mobility on 15% SDS-PAGE. RTA with GST (Lane 2) and RTA alone (Lane 4) were used as blank control; (**c**) ITC binding spectra for GST-C11 titrated to RTA; (**d**) ITC binding spectra for GST-C9 titrated to RTA. The K_D_ value is fitted to a one-site binding model using Origin software (Microcal, Northampton, MA, USA).

RTA can specifically recognize and bind to the C-terminus of human ribosomal stalk protein both in vitro and in vivo to facilitate its ribosome-inactivating activity [10,26]. The C-terminus of P-proteins share a distinctive feature consisting of a cluster of acidic and hydrophobic amino acids, especially the last 11 residues (SDDDMGFGLFD) (as shown in Figure 1a) are conserved. The C-terminus of the stalk protein is functionally significant since it can bind to translation factors both before and after GTP hydrolysis during protein synthesis [11]. The interaction between RTA and P2 was investigated by in vitro pull-down assay. In order to map the boundary sufficient for RTA binding, we fused the last 9, 11, 17 residues of P2 to the GST protein to create GST-C9, GST-C11, GST-C17 fusion proteins, and detected the interactions between RTA and these P2 truncations. Our data showed that RTA could be retained by all three GST-tagged proteins-coupled Sepharose (Figure 1b). The binding affinities were then measured by Isothermal titration calorimetry (ITC). The determined binding affinities of RTA toward C9 and C11 peptide are almost the same, with constant (K_D_) at 3.4 µM and 2.3 µM, respectively (Figure 1c,d).

### 2.2. Structure of RTA-C9-P2 Complex

**Figure 2:**
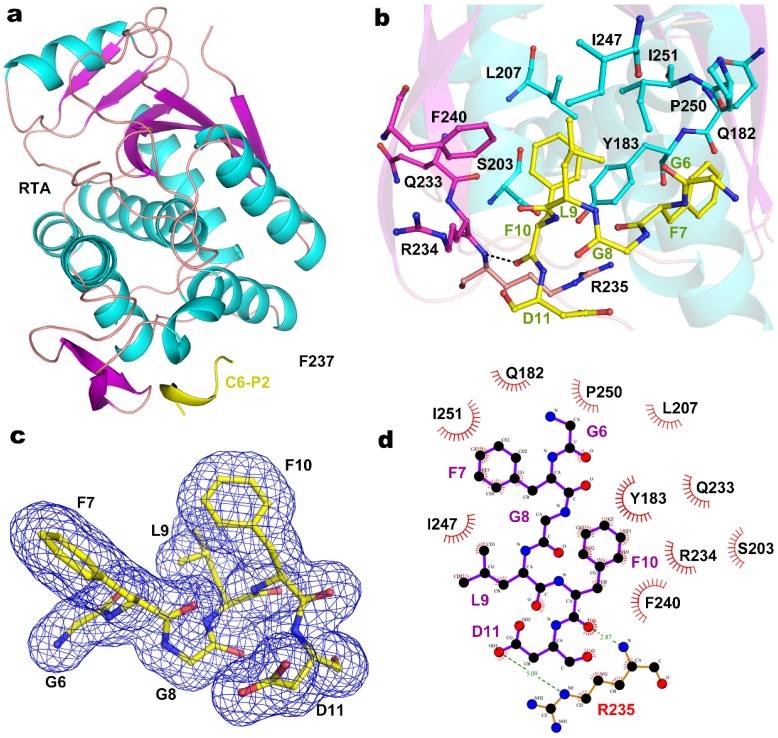
Structure and binding pocket of RTA-C6-P2 complex. (**a**) Overall structure of RTA-C6-P2 complex. The α-helixes, β-sheets and loops of RTA are colored in cyan, magenta and light pink, respectively. Hexameric peptide of P2 ribbon is shown in yellow; (**b**) The binding pocket of C6-P2 peptide. The binding residues are shown as sticks in the same color corresponding to the secondary elements they belong to. The residues of C6-P2 are shown as yellow sticks. The hydrogen bond is indicated by dashed lines; (**c**) 2Fo-Fc electron density map of the P2 peptide at the binding pocket of RTA. The density map is presented as a blue mesh at 1.5 σ level; (**d**) Ligplot analysis of the interactions between RTA and peptide C6-P2.

**Figure 3:**
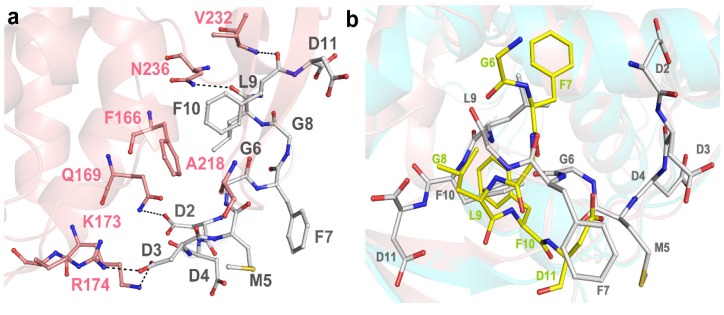
Structural comparison of P2 binding pockets of RTA and TCS. (**a**) P2 binding pocket of TCS-C11-P2 (PDB code: 2JDL). Key residues are colored in pink, and hydrogen bonds are highlighted with black dash; (**b**) Structural superposition of RTA-C6-P2 and TCS-C11-P2. RTA, C6-P2, TCS and C11-P2 are colored in cyan, yellow, pink and gray, respectively.

To further decipher the molecular interaction between RTA and P2, we co-crystallized RTA in complex with a synthetic peptide C11-P2 (SDDDMGFGLFD) and C9-P2 (DDMGFGLFD), respectively. The structure of the RTA-C9-P2 complex and RTA-C11-P2 complex were solved to a resolution of 1.6 Å (Table 1) and 2.3 Å (Table S1), respectively. These two datasets are almost identical, they share the same space group and the RTA molecules and P2 peptides in these two complexes can be superimposed well (Figure S1). Each asymmetric unit of the crystal structure contains two molecules of RTA with an overall root-mean-square deviation (RMSD) of 0.195 Å over 255 Cα atoms. The crystal packing results in a buried interface of 1260 Å^2^ (630 Å^2^/molecule), which was supported by 38 non-bonded contacts, two salt bridges and three hydrogen bonds. Refinement was first performed on RTA, and then C9-P2 peptide was manually built, each molecule of RTA forms a 1:1 complex with C9-P2 peptide (Figure 2a). RTA contains residues 6–260, displaying a highly similar fold as the previously reported structure [27], the overall main chain RMSD of RTA moiety in RTA-C9-P2 complex and wild-type RTA (PDB code: 1RTC) is 0.545 Å over 255 comparable Cα atoms. For C9-P2 and C11-P2 peptide, only the last 6 residues (GFGLFD, C6-P2) could be fitted to the observed electron density, while N-terminal residues DDM and SDDDM were not defined in the final model (Figure 2c). The positions of the two C6-P2 peptide chains are almost identical. The C6-P2 peptide binds to the C-terminal domain of RTA, which is composed of 11 key residues Q182, Y183, S203, L207, Q233, R234, R235, F240, I247, P250 and I251 (as shown in Figure 2b,d), providing a major hydrophobic interface via main-chain and side-chain interactions, while only R235 contributing one key hydrogen bond contact for C6-P2 peptide binding. To further confirm the C6-P2 is sufficient for binding to RTA, we repeated GST-pull down assays using GST-C6, GST-C7 and GST-C8 fusion proteins. By comparison with other P2 truncations, GST-C6, GST-C7 and GST-C8 have similar binding ability (Figure 3a). Using ITC, we determined the RTA binding affinity toward C6, which has a K_D_ of 18.8 µM (Figure 3b), decreasing eight-fold comparing to C11. Using structure modeling (Figure S2), we propose the two positively charged R189 and R193 probably participate in binding to the SDDD motif of P2. Our observations are in contrast to TCS binding wherein a C7 peptide was found unable to bind to TCS [12].

### 2.3. Structural Analysis and Comparison of P2 Binding Pockets of RTA and TCS

**Figure 4:**
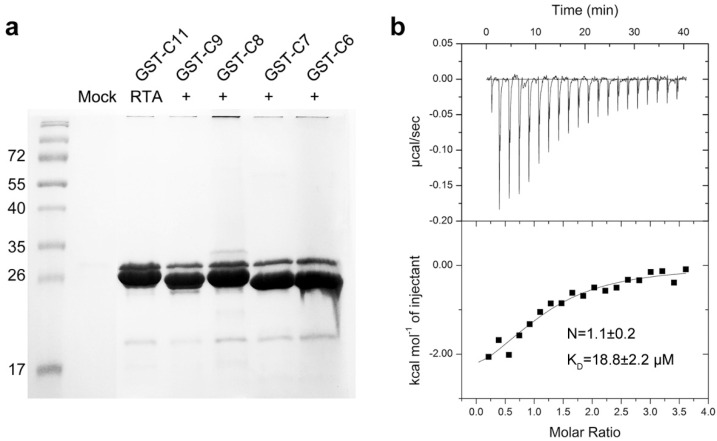
RTA interacts with the conserved C-terminal region of P2. (**a**) The interaction between C6-C8 of P2 and RTA was checked by in vitro GST-pull down assay; (**b**) The ITC spectra of GST-C6 to RTA.

Our previous structural study showed that both the N- and C-terminal regions of C11-P (SDDDMGFGLFD) are involved in the interaction with TCS [12]. The negatively charged DDD motif was stabilized by three hydrogen bonds donated by two positively charged residues K173 and R174, and one non-polar residue Q169 of TCS. The C-terminal hydrophobic region MGFGLFD was stabilized by hydrophobic forces contributed by F166, A218, V232 and N236 (Figure 4a). Four positively charged arginines at the corresponding region in RTA, namely R189, R193, R234 and R235, were identified to be important in RIP-stalk binding [25]. Structural superposition of RTA-C6-P2 and TCS-C11-P2 complex showed RTA and TCS maintain a homologous structure topology (RMSD 1.21 Å over 233 Cα atom) even though they share low similarity in amino acid sequences [28]. However, C6-P2 binds to RTA in a distinguished manner, adopts a different orientation and is seriously distorted when compared with C11-P2 in the TCS-C11-P2 complex (Figure 4b). The P2 binding sites of RTA and TCS are distinctively different, in particularly K173 in TCS, which is the key residue for electrostatic interaction, becomes T190 in RTA. In addition, Q169, V232 and N236 in TCS, which use side-chains for forming hydrogen bonds to accommodate C11-P, become G186, I247 and I251 in RTA, respectively, making hydrogen bonding not plausible. The P2 binding pocket of RTA is hydrophobic and is composed of six hydrophobic residues (Y183, L207, F240, I247, P250 and I251) and five polar residues (Q182, S203, Q233, R234 and R235) to form non-bond contacts to accommodate C6-P2 peptide. Our structural and interaction studies suggested that the conserved hydrophilic DDDM motif at the C-terminus of P2 contributes to interacting with RTA, while the hydrophobic part GFGLFD is accommodated by the hydrophobic binding pocket of RTA. The previous proposed docking model of the RTA-C11 complex [17], has missed some important features, making it not accurate. In the RTA-C6- P2 structure, it revealed that R235 and R234 participate in interacting with F10 and D11 other than interacting with the M5 and G6 in the docking model (Figure S3). Also, most of the hydrophobic residues of RTA, such as Y183, L207, F240, I247, P250 and I251, which contribute to interactions with the hydrophobic part (GFGLF) of P2, were not identified in the docking model (Figure S3b). This also explains why P2 peptide has adopted a different conformation in interacting with RTA.

### 2.4. P2 Protein May Adopt Different Ways of Binding to Different RIPs

**Figure 5:**
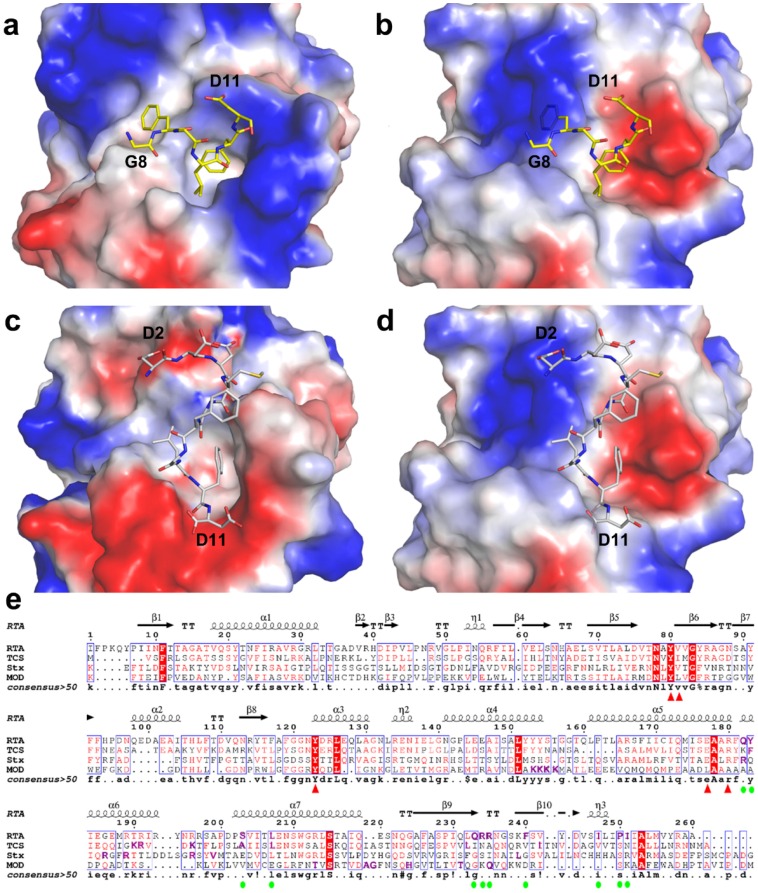
Comparison of surface polarity of P2 binding pocket between RTA and other typical RIPs. (**a**) Electrostatic surface potential map of C6-P2 binding pocket of RTA; (**b**) Electrostatic surface potential map of Stx (PDB code: 1R4Q) using C6-P2 as a reference; (**c**) Electrostatic surface potential map of TCS-C11-P2 (PDB code: 2JDL); (**d**) Electrostatic surface potential map of Stx using C11-P2 as a reference. The surface color represents electric potential, red color is negatively charged surface, blue color is positively charged surface. The neutral region is colored in white or lighter color shade. Electrostatic surface potential is generated by PyMol software; (**e**) Sequence alignment of RTA and other classical RIPs. The key residues of P2 binding pocket of RTA were marked with green dots, whereas the rRNA-glycosidase catalytic residues of RTA were labeled with red triangles. The top secondary structure elements are shown according to the crystal structure of RTA. The identified P2 binding site residues in the different RIPs are highlighted in purple.

From the above structural comparison of the P2 interaction modes and P2 binding site residues of RTA and TCS, we postulate P2 protein may adopt different conformations to interact with RIPs to facilitate RIPs gaining access to ribosome. This is supported by the different charge distribution patterns on RIPs. We superimposed the structures of MOD (PDB code: 2PQJ) and Stx (PDB code: 1R4Q) onto RTA and TCS individually to compare their putative P2 binding pockets. These RIPs are structurally homologous to each other, Stx and MOD possess a similar fold as RTA with RMSD 1.95 Å over 210 Cα atoms and 2.08 Å over 195 Cα atoms, respectively. Meanwhile, compared with TCS, Stx exhibits a lower RMSD 1.21 Å over 233 comparable Cα atoms than MOD with a RMSD 1.89 Å over 203 comparable Cα atoms. The overall landscape and polarity of the electrostatic surface potential around P2 binding pockets of RTA (Figure 5a) and TCS (Figure 5c) are quite different, which probably determined the orientation of P2 peptide upon binding. We attempted to predict P2 binding modes on Stx by superimposing C6-P2 and C11-P2 to Stx. Compared with TCS and RTA, the P2 binding pocket of Stx exhibited distinct shape and electrostatic charge distributions using C6-P2 and C11-P2 as reference (Figure 5b,d). Based on the complex structure of TCS-C11-P2 and RTA-C6-P2, we mapped the related residues of the hydrophobic patch within 4 Å from C11-P2/C6-P2 in TCS (F166, A184, L188, L215, N216, V223 and L225) and RTA (Y183, L207, F240, I247, P250 and I251). The amino acid composition and configuration were found to be different. At the corresponding position, we identified six residues S225, F226, G227, N230, A231 and G234 in Stx, which are also distinct from the corresponding residues in TCS and RTA. For the positively charged patch comparison, R176 and R179, K173 and R174, R189 and R193 were found in Stx, TCS and RTA, respectively. Although the distance between each pair is not the same, they may form charge-charge interactions with the DDD motif of P2. The different shape, electrostatic charge distribution of the P2 binding pocket and variant composition of hydrophobic patch suggested these RIPs possibly using a novel binding mode to interact with P2.

In addition, we previously determined that MOD interacts with the conserved C-terminal peptide of P2 without hydrophobic interactions [29], in a manner different to TCS or RTA. Four positive charged lysines K143-K146 were identified to be involved in matching the negative charged DDD motif on P2 [14]. They are located far from the positively charged region which corresponds to binding the acidic DDD motif on TCS (corresponding residues K173, R174 and K177). Comparison of primary sequences shows that these identified amino acid residues responsible for P protein interaction in Stx and MOD are not conserved in TCS and RTA (Figure 5e). The variability of composition of P2 binding site residues, pocket shape and charge distribution of RIPs, implying that C-terminal region of P protein adopts a flexible conformation to interact with different RIPs.

## 3. Discussion

In this study we have solved the structure of RTA complexed with C6-P2 peptide. In this structure, a hydrophobic pocket at the C-terminal domain of RTA donates a major interface to associate with the hydrophobic GFGLFD motif on the human ribosomal stalk P2 peptide. Nevertheless, our in vitro interaction assays also showed that the negatively charged DDD motif on P2 peptide does contribute to the binding of RTA (K_D_ of RTA and C11-P2 is 2.3 µM, C6-P2 is 18.8 µM). Using structure modeling, we identified two arginines, R189, R193 probably interact with the N-terminal DDD motif of P2. This is consistent with previous biochemical data which identified four positively charged arginines R189, R193, R234 and R235 on RTA to be important in RIP-stalk binding [25].

Our structure of RTA-C9-P2 only captured the electron density of C6-P2; this may be caused by the flexibility of the N-terminal of P2, which was also observed in our previous structural study of TCS-C11-P2 complex. The asymmetric unit contains two molecules of TCS and two C11-P2 peptide (chain C and D). For chain C, C10-P2 (DDDMGFGLFD) could be fitted to the observed electron density and for chain D, only C9-P2 (DDMGFGLFD) was fitted [17]. A possible explanation is that RIPs form transient complexes with ribosomal stalk P proteins to facilitate their fast access to the targeted adenine on the SRL of the ribosome. It was previously evidenced that the interaction of RIP with ribosomal protein increased the efficiency of the rat ribosome inactivation activity of RTA, with a catalytic efficiency is 80,000 times faster than that on naked 28S rRNA [6]. The current structure may only represent a snapshot of a series of transient interactions in the course of SRL targeting. Even though we demonstrated hydrophobic interactions play an important role in mediating RTA and ribosomal stalk P protein interaction, electrostatic interactions might be involved in the subsequent proximity steps to the SRL of ribosome, as a previous study mentioned electrostatic interactions might be exploited by RTA to facilitate ribosome targeting in a similar mechanism of restrictocin [30].

According to the binding mode of P2 peptide to TCS and RTA, the shape and key residues of P2 binding pocket in the RIP may affect the P2 conformation. The structural differences implied that the C-terminus of P2 may as well adopt a flexible conformation for binding. Our previous studies also have shown that MOD interacts with the conserved C-terminal P2 peptide via charge-charge interactions but not hydrophobic interactions and the binding pocket in MOD may be far away from those of RTA and TCS [29]. Comparison of the primary sequences shows that the biochemically identified amino acid residues responsible for P protein interaction in Stx and MOD are not conserved in other RIPs (Figure 5e).

In sum, we demonstrate the amino acid composition, shape and polarity of P2 binding pocket of RIPs are versatile. This strengthened our hypothesis that P2 protein may adopt different conformation and orientation to bind RIP.

## 4. Materials and Methods

### 4.1. Cloning, Expression, and Purification of RTA and P2 Variants

RTA gene was cloned into PET28a with a hexahistidine tag at the N-terminus. The *Escherichia coli* BL21 (DE3) pLysS strain was used for the expression of recombinant protein. The transformed cells were grown at 37 °C in LB culture medium (Invitrogen, Life Technologies, Camarillo, CA, USA) containing appropriate antibiotics until the OD_600 nm_ reached about 0.8. Protein expression was then induced with 0.2 mM isopropyl 1-thio-β-d-galactopyranoside (Sigma-Aldrich, St. Louis, MO, USA) by another 20 h at 16 °C. Cells were harvested by centrifugation (6000× *g*, 4 °C, 10 min) and resuspended in 40 mL of lysis buffer (50 mM Tris-Cl, pH 7.5, 150 mM NaCl, 5% (*v*/*v*) glycerol). After cell disruption and centrifugation, the supernatant containing the soluble target protein was purified with a HisTrap HP 5 mL column (GE Healthcare Biosciences, Pittsburgh, PA, USA) equilibrated with the binding buffer (20 mM Tris-Cl, pH 7.5, 150 mM NaCl). The target protein was eluted and further purified by Superdex 75 column (GE Healthcare Biosciences, Pittsburgh, PA, USA) equilibrated with 20 mM Tris-Cl, pH 7.5, 100 mM NaCl. Protein purity was evaluated by SDSPAGE, and concentrated to appropriate concentration by ultrafiltration (Millipore Amicon, Merk, Darmstadt, Germany). After liquid nitrogen freezing, protein samples were stored at −80 °C.

The last 6, 7, 8, 9, 11 and 17 residues of P2 were cloned into PGEX-4T-1 vector (GE Healthcare Life Sciences, Pittsburgh, PA, USA) with a GST-tag at the N-terminus. GST and the recombinant GST-tagged proteins were purified by glutathione sepharose chromatography (GE Healthcare Biosciences, Pittsburgh, PA, USA) and gel filtration individually using PBS pH 7.4 buffer (USB, Cleveland, OH, USA). Purity was assessed by SDS-PAGE and protein stored in the same manner as RTA.

### 4.2. Crystallization, Data Collection and Processing

Synthesized peptides C9-P2 and C11-P2 (GL Biochem, Shanghai, China) were added into RTA to final concentration 5 mM and incubated for 6 h at 4 °C before crystallization.

Commercially available Crystal Screen 1–2 and Index screen (Hampton Research) were used for crystallization trials in 96-well plates (XtalFinder, XtalQuest Inc., Beijing, China) at 16 °C. The crystals were obtained using the hanging drop vapor-diffusion method, by equilibrating 1 μL of 15 mg/mL RTA-C9-P2 mixture with an equal volume of the reservoir solution (2.8 M sodium acetate, tetrahydrate pH 7.0) (USB, Cleveland, OH, USA). Further optimization was carried out using Additive Screen kit (Hampton Research). Crystals which produced good diffraction quality were grown in 2.8 M sodium acetate tetrahydrate, pH 7.0, 30%–35% glucose. All the crystals were transferred to cryoprotectant (reservoir solution supplemented with 30% glycerol) and flash-cooled with liquid nitrogen. The data were collected at 100 K in a liquid nitrogen stream using beamline 13B1 with a Q315r CCD (ADSC, MAR Research, Norderstedt, Germany) at the Biological Crystallization Facility at National Synchrotron Radiation Research Center (NSRRC), Hsinchu, Taiwan. Data were scaled and merged with ScalePack installed with HKL2000 [31].

### 4.3. Structure Determination and Refinement

The structure of the RTA-C6-P2 complex was determined by molecular replacement with Phaser in CCP4 suite [32] using Protein Data Bank code 3PX8 as the search model. The initial model of the RTA was obtained and refined by REFMAC5 [33]. The C9-P2 peptide was manually built and refined in Coot [34]. The overall assessment of model quality was evaluated using the programs MOLPROBITY [35] and PROCHECK [36]. Sequence alignment was prepared using the online program Multalin (F.Corpet, INRA Toulouse, France) (http://multalin.toulouse.inra.fr/multalin/). The crystallographic parameters of the structure are listed in Table 1. All structure figures were prepared with PyMOL (DeLano Scientific, Palo Alto, CA, USA) [37].

### 4.4. In Vitro GST-Pull-Down Assays

To assess the interaction of RTA with GST-P2 variants, 45 µL 60 µM RTA was added into 50 µL 35 µM GST fusion P2 variants, incubated for 30 min at 4 °C. 20 µL pre-equilibrated glutathione-affinity resin was added into the system and then incubated for another 30 min. Then the beads were washed thoroughly by PBS pH7.4 buffer twice. Finally, the bound protein was eluted from beads with 20 µL 10 mM reduced glutathione. RTA alone was used as control. All the samples were detected by 15% SDS-PAGE.

### 4.5. Isothermal Titration Calorimetry (ITC)

The binding affinities of RTA toward P2 variants were determined by ITC-200 (Microcal, Northampton, MA, USA). All protein solutions were degassed without stirring and kept on ice before ITC experiments. Titrations were performed at 25 °C in buffer containing 20 mM Tris-Cl, 100 mM NaCl, pH 7.0 by injecting 20 consecutive aliquots (2 µL) of 600 µM P2 variant peptides solution into the ITC cell (200 µL) containing 35 µM RTA solution, using P2 variant peptides titrated into buffer as blank control. The data were collected and analyzed using Origin software (Microcal, Northampton, MA, USA). Thermodynamic parameters were determined using nonlinear least squares fitting, assuming a one-site binding model.

## Figures and Tables

**Table 1:** Crystal parameters, data collection, and structure refinement.

RTA-C6-P2	
Data collection	
Space group	*P 1 21 1*
Unit cell *a*, *b*, *c* (Å) α, β, γ (°)	67.45, 59.88, 67.50 90.00, 99.89, 90.00
Resolution range (Å)	25.00–1.55 (1.61–1.55) ^a^
Unique reflections	76937
Completeness (%)	97.8 (96.4)
<*I/σ(I)*>	24.0 (3.5)
R_merge_^b^ (%)	33.9 (4.6)
Average redundancy	3.6 (3.6)
Structure refinement	
Resolution range (Å)	24.65–1.55
R-factor ^c^/R-free ^d^ (%)	25.6/21.7
Number of protein atoms	4366
Number of water atoms	234
RMSD ^e^ bond lengths (Å)	0.0261
RMSD bond angles (°)	1.500
Ramachandran plot ^f^ (residues, %)	-
Most favored (%)	95.8
Additional allowed (%)	4.2
Outliers (%)	0
PDB entry	5GU4

^a^ The values in parentheses refer to statistics in the highest bin; ^b^ R_merge_ = ∑_hkl_∑*_i_*|*I_i_*(hkl) − <*I*(hkl)> |/∑_hkl_∑*_i_I_i_*(hkl), where *I_i_*(hkl) is the intensity of an observation and <I(hkl)> is the mean value for its unique reflection; Summations are over all reflections; ^c^ R-factor = ∑_h_||*F_o_*(h)| − |*F_c_*(h)||/∑_h_|*F_o_*(h)|, where |*F_o_*| and |*F_c_*| are the observed and calculated structure-factor amplitudes, respectively; ^d^ R-free was calculated with 5% of the data excluded from the refinement; ^e^ Root mean square deviation from ideal values; ^f^ Categories were defined by Molprobity.

## Supplementary Materials

The following are available online at www.mdpi.com/2072-6651/8/10/296/s1, Figure S1: Superposition of the structure of RTA-C9-P2 and RTA-C11-P2, Figure S2: The putative binding model of RTA and C11-P2, Figure S3: Structural superposition of RTA-C6-P2 with previous docking model, Table S1: Crystal parameters and data collection of RTA –C11-P2.
