# Supplementary material for: Crystal Structure of Ribosome-Inactivating Protein Ricin A Chain in Complex with the C-Terminal Peptide of the Ribosomal Stalk Protein P2
Source: Toxins (Basel). 2016 Oct 13;8(10):296. doi: 10.3390/toxins8100296 (PMC5086656; doi:10.3390/toxins8100296)
Supplement: Supplementary file 1 [file toxins-08-00296-s001.pdf]

Supplementary Materials: Crystal Structure of Ribosome-Inactivating Protein Ricin A Chain in Complex with the C-Terminal Peptide of the Ribosomal Stalk Protein P2

Wei-Wei Shi, Yun-Sang Tang, See-Yuen Sze, Zhen-Ning Zhu, Kam-Bo Wong and Pang-Chui Shaw

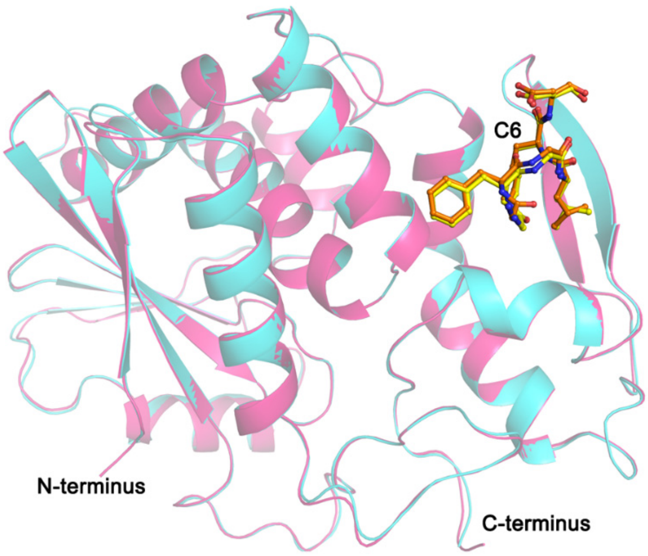

Figure S1. Superposition of the structure of RTA-C9-P2 and RTA-C11-P2. The RTA molecule and C6 peptide can be well superimposed, with an overall RMSD of 0.33 Å over 255 Cα atoms. The RTA molecule and C6 portion of RTA-C9-P2 are colored in cyan and yellow, the corresponding RTA and C6 portion of RTA-C11-P2 are colored in magenta and orange, respectively.

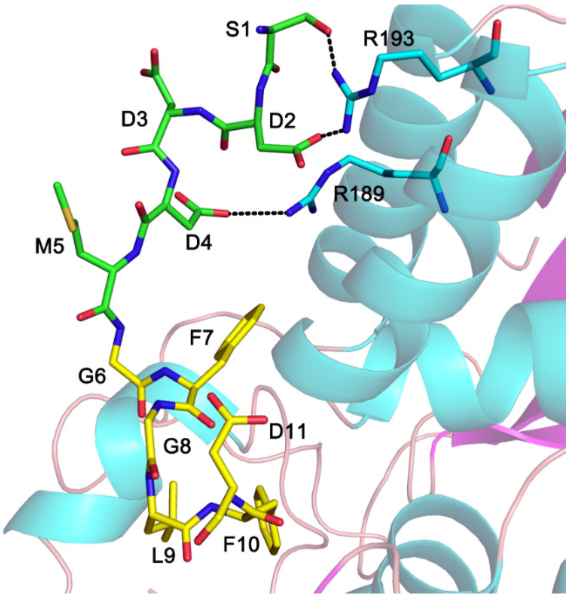

Figure S2. The putative binding model of RTA and C11-P2. The *N*-terminal SDDDM residues were manually built along to the trend of C6-P2 peptide chain in coot software [1]. These residues are shown in green sticks. Hydrogen bonds are highlighted with black dash.

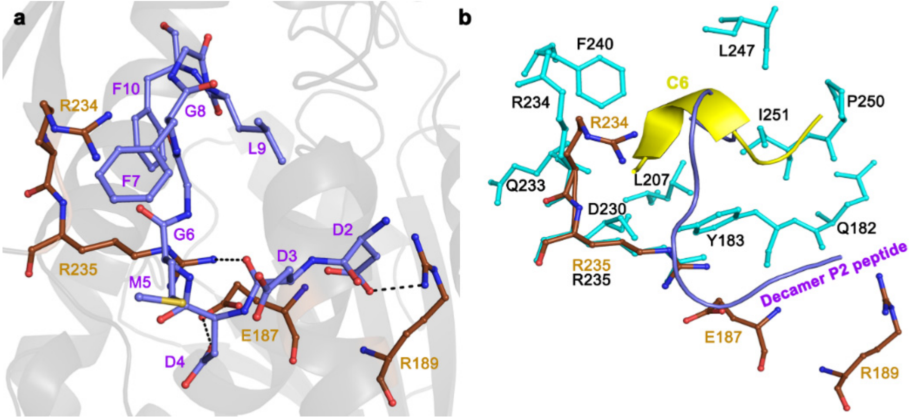

Figure S3. Structural superposition of RTA-C6-P2 with previous docking model. (**a**) Previous docking model of RTA and decamer P2 peptide (DDDMGFGFLF) [2]. RTA molecule is colored in gray. The decamer P2 peptide is shown as blue stick, and the proposed key P2 peptide binding residues are shown as brown sticks; (**b**) The structural superposition of C6-P2 in the present crystal structure with decamer P2 peptide in previous docking model shows C6-P2 adopts a different orientation compared to the decamer P2 peptide. C6-P2 peptide of RTA-C6-P2 crystal complex is shown as yellow stick and the decamer P2 peptide in the docking model in blue. Our structurally identified P2 peptide binding residues in RTA are shown as cyan sticks.

Table S1. Crystal parameters and data collection of RTA –C11-P2.

**RTA–C11-P2**	
Data collection	
Space group	*P 1 21 1*
Unit cell<br>*a*, *b*, *c* (Å)<br>*α*, β, *γ* (°)	67.06, 60.48, 68.13<br>90.00, 99.89, 90.00
Resolution range (Å)	44.93–2.3 (2.35–2.30) [a]
Unique reflections	34724
Completeness (%)	98.8 (97.6)
$<I/\sigma(I)>$	24.0 (3.5)
R_{merge} [b] (%)	27.9 (3.0)
Average redundancy	3.2 (3.3)

[a] The values in parentheses refer to statistics in the highest bin; [b] $R_{merge} = \sum_{hkl}\sum_i |I_i(hkl) - <I(hkl)>| / \sum_{hkl}\sum_i I_i(hkl)$, where I_i(hkl) is the intensity of an observation and <I(hkl)> is the mean value for its unique reflection; Summations are over all reflections.

References

1. Emsley, P.; Cowtan, K. Coot: Model-building tools for molecular graphics. *Acta Crystallogr. D Biol. Crystallogr.* **2004**, *60*, 2126–2132.
2. Too, P.H.; Ma, M.K.; Mak, A.N.; Wong, Y.T.; Tung, C.K.; Zhu, G.; Au, S.W.; Wong, K.B.; Shaw, P.C. The C-terminal fragment of the ribosomal P protein complexed to trichosanthin reveals the interaction between the ribosome-inactivating protein and the ribosome. *Nucleic Acids Res.* **2009**, *37*, 602–610.
